# Effects of Bile Acids on Growth Performance, Hepatopancreatic Antioxidant Capacity, Intestinal Immune-Related Gene Expression, and Gut Microbiota of *Penaeus vannamei*

**DOI:** 10.3390/ani15020240

**Published:** 2025-01-16

**Authors:** Yun Zhao, Duanduan Chen, Hui Wang

**Affiliations:** 1 College of Animal Science and Technology, Shandong Agricultural University, Taian 271018, China; yunzhao0503@foxmail.com; 2 College of Agriculture and Biology, Liaocheng University, Liaocheng 252000, China

**Keywords:** *Penaeus vannamei*, bile acids, antioxidant, immune, gut microorganisms

## Abstract

This study aimed to examine the impact of varying concentrations of bile acids (BA) added to the feed on several aspects of *Penaeus vannamei*. The purity of BA was 25.29%, and its main components were 5.74% chenodeoxycholic acid, 6.27% allocholic acid, 3.20% cholic acid, 5.79% hyodeoxycholic acid, and 2.31% hyocholic acid. The experiment was designed with four groups: CT, BA1, BA2, and BA3, where BA were added to the shrimp basal diet at concentrations of 0.0 mg/kg, 0.1 mg/kg, 1.0 mg/kg, and 10.0 mg/kg, respectively. After 60 days of farming *P. vannamei* (initial body weight: 1.21 ± 0.05 g), the results showed that BA supplementation significantly improved growth performance, and BA2 group was the most significant, which increased the final weight (FBW) by 18.6%, weight gain rate (WGR) by 19.5%, and survival rate (SR) by 5.8% compared with the CT group (*p* < 0.05). Additionally, the activities of trypsin and lipase in gut tissue were significantly increased (*p* < 0.05). Furthermore, BA supplementation increased the activity of antioxidant-related enzymes in the hepatopancreas and enhanced the mRNA expression levels of gut-associated immune genes. In addition, the supplementation of 0.1 mg/kg BA significantly altered the gut microbial composition, reducing the proportion of harmful Proteobacteria while enhancing the relative abundance of beneficial microorganisms such as Firmicutes and Bacteroides. In conclusion, 1.0 mg/kg and 10.0 mg/kg BA supplementation significantly improved the growth performance, digestive capacity, and antioxidant capacity of shrimp, among which 1.0 mg/kg supplementation had the most significant effect and improved the intestinal microbial composition of shrimp.

## Simple Summary

This study examines the effects of adding bile acid (BA) to shrimp diets and how this affects the growth and health of *Penaeus vannamei*. The study involved 360 shrimp fed either a basal diet or a BA-supplemented diet, and monitored shrimp growth, survival, and microbial community structure. The results showed that BA supplementation was beneficial to the growth of shrimp and increased the activity of intestinal digestive enzymes, hepatopancreas antioxidant enzymes, and the mRNA expression levels of gut-related immune genes. It also significantly changed the composition of gut microbes, reducing the proportion of harmful Proteobacteria and increasing the relative abundance of beneficial microorganisms such as Firmicutes and Bacteroides. These findings suggest that dietary supplementation with BA improves the health of shrimp and promotes growth and development, which may be a way to promote the growth of shrimp.

## 1. Introduction

*Penaeus vannamei* (*Litopenaeus vannamei*), a species belonging to the family Penaeidae and genus *Penaeus*, is the most widely farmed shrimp species in the aquaculture industry and serves as a key source of high-quality protein for humans [1]. Currently, the global production of *P. vannamei* is steadily increasing [2]. In order to meet the growing demand, common poor management practices, such as increased stocking densities and excessive overfeeding, have become prevalent. These practices can lead to nutritional imbalances in farmed shrimp and the deterioration of the aquaculture environment. They not only increase the burden on the shrimp’s hepatopancreas but also make the shrimp more susceptible to diseases. In recent years, the addition of functional additives to feed has emerged as an effective strategy to improve shrimp health [3,4]. Specifically, natural bioactive substances extracted from plants and animals are recognized as important functional supplements with great potential for aquaculture due to their simple, safe biodegradation properties [5].

Bile is the primary pathway for the excretion of toxins in vertebrates, and it is composed of bile acids (BA). After being metabolized and transformed in the liver, toxins and their metabolites can be excreted from the body through BA [6]. Therefore, BA can be considered detergents for toxins in vertebrates. They are a group of alcohols produced from cholesterol metabolism in the liver of both humans and animals. BA exist in the form of bile salts under physiological conditions, with high activity, good water solubility, and weakly acidic. They are primarily found in the liver and intestinal circulatory system, where they exert protective effects on the body [7,8]. These protective effects include promoting the digestion and absorption of intestinal cholesterol, triglycerides, fatty acids, and fat-soluble vitamins [9,10], regulating the feedback regulatory mechanisms of BA biosynthesis in the liver and gallbladder motor function [11]. The gut–liver axis also plays a key role in the response to inflammation [12], such as immune responses [13,14,15], epithelial cell proliferation [16], gut microbes [17], and gene expression through epigenetic mechanisms [18]. It can also regulate intestinal cholesterol excretion and reverse cholesterol transport [19]. As invertebrates, crustaceans cannot synthesize BA because they lack the ability to synthesize cholesterol through the liver from acetate and mevalonate [20,21]. Therefore, BA can only be obtained through diet. Since the 1980s, BA have been added to aquatic feeds, where they have played a crucial role in enhancing lipid digestion and absorption [21]. In recent years, there have been many studies on the functions and applications of BA, demonstrating that supplementing diets with BA can significantly benefit species such as paralichthys olivaceus (*Temminck and Schlegel*) [22], large yellow croaker (*Larimichthys crocea*) [23], largemouth bass (*Micropterus salmoides*) [24], Nile tilapia (*Oreochromis niloticus*) [25], tiger puffer (*Takifugu rubripes*) [26], grass carp (*Ctenopharyngodon idella*) [27], and others. These benefits include improved growth, enhanced feed conversion efficiency, better liver function, increased digestive enzyme activity, and reduced stress responses. It has also been shown that BA can also contribute to the development of a healthy diet and that they also help white shrimp (*Litopenaeus vannamei*) molt [28]. At the same time, due to the lack of reabsorption of BA by the enterohepatic circulatory system, it is hypothesized that the toxin excretion of BA in invertebrates may be more efficient than in vertebrates [29].

Gut microorganisms are essential for supporting nutrient digestion and absorption, as well as for regulating the physiological functions of the gut [30]. Gut microbiota components are regulated by extracellular metabolites from the host and modified by the microbes. Among them, BA represent highly rich reservoirs of host-derived and microbially modified metabolites that are major regulators of the gut microbiome [31]. Specifically, BA can shape the gut microbiome by promoting the growth of BA-metabolizing bacteria and inhibiting the growth of other bile-sensitive bacteria [32,33]. In addition, gut microorganisms are important for the metabolism of BA in the organism, aiding in the conversion of primary BA to secondary ones, enhancing their diversity, and facilitating their excretion in feces [34]. Earlier research has indicated that BA can attenuate the detrimental effects of mixed plant protein sources on the gut microbiota of Amur sturgeon (*Acipenser schrenckii*) [35]. Overall, BA are functional feed additives that can improve the growth and health of shrimp.

Based on this background, the present study investigates the effects of BA supplementation in feed on the growth performance, gut digestibility, immunity, hepatopancreatic antioxidant capacity, and gut microbial composition of *P. vannamei*. This includes assessing the effects on growth performance such as survival, weight increase, and feed conversion; investigating the effects on intestinal digestive ability, including amylase, trypsin and lipase; and analyzing the effects on intestinal immunity. To evaluate the effects on the antioxidant capacity of hepatopancreas and measure the activity of antioxidant enzymes (such as SOD, CAT and GSH-PX), the effects of changes in intestinal microbial community composition were investigated by high-throughput sequencing. These studies are expected to deepen our understanding of the nutritional significance of BA in aquatic products, especially crustaceans, and provide scientific basis for its practical application in aquaculture.

## 2. Materials and Methods

### 2.1. Experimental Materials and Shrimp

The basic feed for the shrimp used in the experiment was supplied by Shandong Delta Bio-conversion Co. Ltd., Shandong, China, with the specific raw materials and nutritional composition detailed in Table 1. The BA used in the test were provided by Shandong Zhongjing Biotechnology Co. Ltd., Shandong, China, with a purity of 25.29%. The active ingredients of the BA included 5.74% chenodeoxycholic acid, 6.27% allocholic acid, 3.20% cholic acid, 5.79% porcine deoxycholic acid, and 2.31% porcine bile acid. The test animals, *P. vannamei*, were supplied by Rizhao Xinhui Aquatic Seedling Co. Ltd., Shandong, China and were temporarily housed in the laboratory until they reached the juvenile stage (1.21 ± 0.05 g), at which point the experiment commenced.

### 2.2. Experimental Design and Feeding Management

A total of 360 healthy, uniformly sized juvenile *P. vannamei* with similar body weight (1.21 ± 0.05 g) and normal feeding were selected and randomly assigned to four groups, CT, BA1, BA2, and BA3. Each group included three replicates, with 30 shrimp in each replicate. Each replicate was reared in a circular tank containing 140 L of water and equipped with a device that facilitates oxygenation. During the culture period, the hens in the CT group were fed a basal diet, and the BA1, BA2, and BA3 groups were fed the basal diet supplemented with BA at concentrations of 0.1, 1.0, and 10.0 mg/kg, respectively. Before each feeding, different concentrations of BA were manually mixed with the base feed. The shrimp in each group were fed experimental diets with 10–15% of their body weight at regular intervals (9:00, 15:00, and 19:00) every day, and the residual bait feces were extracted in time. The water quality was tested regularly during the breeding period. The water quality was maintained within optimal ranges. The temperature was maintained at 25 ± 1 °C, dissolved oxygen levels concentration was higher than 5 mg/L, the pH was 8.0 ± 0.2, the salinity was 2.0–3.0‰, ammonia nitrogen concentration was lower than 0.01 mg/L, and nitrite levels were kept under 0.1 mg/L. The water was changed regularly to ensure the healthy growth of the shrimp.

### 2.3. Measurement of Growth Parameters and Sample Acquisition

After 60 d of cultivation, all the *P. vannamei* were counted and weighed to assess growth performance, including survival rate, body weight growth rate, feed intake, and feed conversion rate. The formulas used for these calculations were as follows:

Survival rate (SR) (%) = final trail/initial trail × 100%,(1)

Weight gain rate (WGR) (%) = (Final weight − initial weight)/initial weight × 100,(2)

FI (feed intake, g/shrimp) = (dry diet given − dry remaining diet recovered)/number of shrimp(3)

Food conversion ratio (FCR) = dry feed/weight gain(4)

After counting and weighing, three shrimps from each group were randomly selected to collect their hepatopancreas and gut tissues. These samples were placed in cryovials, rapidly frozen in liquid nitrogen, and stored at −80 °C for future analysis.

### 2.4. Measurement of Digestive Enzyme Activity in the Gut and Antioxidant Enzyme Activity in the Hepatopancreas

Hepatopancreas and gut tissue samples were homogenized on ice with 0.9% saline at the ratio of weight (g) to volume (mL) = 1:9. The resulting mixture was then centrifuged at 3000 rpm for 10 min, and the supernatant was collected for further analysis. The activities of amylase, lipase, and trypsin in the gut tissue were measured using assay kits (C016-1-1, A054-1-1, A080-2) from Nanjing Jiancheng Institute of Bioengineering. Amylase activity was measured using colorimetry, which detects the breakdown of starch into reducing sugar, and the amount of color change is directly related to the activity of the enzyme. Trypsin activity can be determined by quantifying it using spectrophotometry, in which the enzyme cleaves a specific substrate to release the chromogenic product. Lipase activity was measured by monitoring the hydrolysis of triglycerides to free fatty acids and glycerol. Additionally, the Hepatopancreas’s superoxide dismutase (SOD), catalase (CAT), and glutathione peroxidase (GSH-PX) activity, as well as malondialdehyde (MDA) content, were assessed using the respective assay kits (A001-1, G4307, A005, G4300) from the same institute. The concentration of free fatty acids was determined by colorimetry. Superoxide anion radical (O_2_^−^) was produced by the reaction system of xanthine and xanthine oxidase. The latter oxidized hydroxylamine to form nitrite, which presented a purplish red color under the action of chromogenic agent. Its absorbance was measured by visible light spectrophotometer. The SOD activity in the measured sample was calculated by the formula. CAT activity was assessed by measuring the rate of hydrogen peroxide (H_2_O_2_) decomposition, and the decrease in H_2_O_2_ concentration over time was monitored using spectrophotometry. The GSH-PX activity was determined by the rate at which glutathione (GSH) was oxidized in the presence of hydrogen peroxide, and the level of oxidized glutathione was measured by spectrophotometry. MDA is one of the products of lipid peroxidation and can react with thiobarbiturate (TBA) to form brownish-red complex under high temperature conditions, MDA levels are quantified by measuring absorbance or fluorescence intensity to assess lipid peroxidation levels in cells or tissues. The protein concentration of the sample was determined by the BCA protein concentration determination kit (Beijing Solarbio Technology Co., Ltd., Beijing, China), under alkaline conditions. The protein reduced Cu_2_^+^ to Cu^+^, and Cu^+^ formed a purply-blue complex with the BCA reagent. The absorption value at 562 nm was determined and compared with the standard curve to calculate the concentration of the protein to be measured. The methods of operation for the determination of all indicators were performed following the manufacturer’s instructions provided with each kit.

### 2.5. Real-Time Quantitative PCR (qRT-PCR) Detection

Total RNA was extracted from the shrimp gut tissue using an RNA extraction kit for aquatic animal (Tiangen Biotechnology Co., Ltd., Beijing, China). RNA quality was assessed by measuring concentration and integrity through 1% agarose gel electrophoresis and Thermo Scientific™ NanoDrop™ One U.S.A. Ultra-Micro UV spectrophotometer. Next, the RNA was quantified to 1 μg, and cDNA was synthesized using the Evo M-MLV RT Kit (Hunan Eric Bioengineering Co., Ltd., Hunan, China) according to the manufacturer’s protocol. Gene-specific primers for qRT-PCR were designed using Primer 5 software and synthesized by Shanghai Shenggong Biological Engineering Co., Ltd., Shanghai, China. The primer sequences are listed in Table 2, including primer names and their corresponding sequences. After confirming the specificity of the primers and the suitability of the cDNA templates, fluorescence-based qRT-PCR was performed using the SYBR^®^ Green Premix Pro Taq HS qPCR Kit (Precision Biotechnology Ltd., Wuhan, China), following the kit’s instructions. β-actin served as the internal reference gene for mRNA expression analysis, and the 2^−ΔΔCT^ method was used for data calculation and statistical analysis. Before the data analysis, we conducted a preliminary normality test on the CT values of the samples, and the results showed that the data corresponded to the normal distribution.

### 2.6. Analysis of Differential Microorganisms in the Gut

According to the instructions of the bacterial DNA extraction kit (Tiangen Biotechnology Co., Ltd., Beijing, China), the total bacterial DNA in the intestinal samples of CT and BA2 groups (n = 3) was extracted. The concentration and purity of DNA were measured using 1% agarose-gel electrophoresis and NanoDrop 2000 (Thermo Fisher Scientific Co, Ltd., Shanghai, China), and the sample was diluted to 1 ng/μL with sterile water. According to the Phusion^®^ High-Fidelity PCR Master Mix with GC Buffer (New England Biolabs Co., Ltd., Beijing, China) instructions, the bacterial universal primers (338F: ACTCCTACGGGAGGCAGCAG and 806R: GGACTACHVGGGTWTCTAAT) amplified the V3-V4 region of the bacterial 16S rRNA gene. PCR products were recovered using a universal DNA purification recovery kit (Tiangen Biotechnology Co., Ltd., Beijing, China). The libraries were constructed using the NEBNext^®^ Ultra™ II DNA Library construction Kit and sequenced using NovaSeq6000 (2 × 250 bp). Sequencing data were processed using FLASH software (V1.2.11, http://ccb.jhu.edu/software/FLASH/, accessed on 13 October 2023) to join the data obtain the original Tags data (Raw Tags). Then, fastp software was used for quality control to obtain high-quality Clean Tags. The Tags obtained after the above processing need to be processed to remove the chimeric sequence. The Tags sequences were compared with the species annotation database (silva database https://www.arb-silva.de/, accessed on 13 October 2023) to detect the chimeric sequence, and the chimeric sequence was finally removed to obtain the final Effective Tags. The DADA2 module of QIIME2 software was used to filter sequences with abundance less than five, and the final ASV (Amplicon Sequence variant) and feature list were obtained. Subsequently, QIIME2 (http://qiime.org, accessed on 13 October 2023) was used in combination with the green gene database for noise reduction and comparison analysis of high-throughput sequencing data. Bacterial community composition was comprehensively analyzed to obtain specific information on each ASV. LEfSe analysis (difference screening threshold LDA score > 4) was used to screen the different species between groups.

### 2.7. Data Statistics and Analysis

SPSS 22.0 software (IBM Corporation, Armonk, NY, USA) was used to perform one-way ANOVA on the data. Shapiro–Wilk normality test was used to evaluate the normality of each dataset before applying the parameter test, and variance homogeneity test was used to evaluate the homogeneity of the variance using Levene test. To avoid class I errors due to multiple comparisons, we applied Tukey’s correction when performing post-multiple comparisons between groups. Histograms were plotted using GraphPad Prism 8.0 (GraphPad Software Inc., San Diego, CA, USA). The findings were reported as means ± standard deviation (SD) in tables and figures (n = 3). The level of significance was set at a *p*-value (*p*) < 0.05.

## 3. Results

### 3.1. The Effect of Adding BA to Feed on the Growth Performance of P. vannamei

As can be seen in Table 3, the effect of adding BA to feeds of *P. vannamei* was that their the growth performance improved following 60 days of BA supplementation. The final body weight (FBW) and weight gain rate (WGR) in the BA2 group were increased by 18.6% and 19.5%, respectively, which are significantly higher than those in the CT group (*p* < 0.05). Compared to the CT group, BA supplementation also slightly increased feed intake (FI) but decreased feed conversion ratio (FC), although these differences were not statistically significant. Notably, the inclusion of BA also increased the survival rate (SR) of the shrimp: the SR was significantly higher in the BA2 and BA3 groups compared to the CT group (*p* < 0.05), increasing by 5.8% and 5.4%, respectively. Observations indicated that WGR, FI, and SR of prawns increased with higher BA concentrations in the diet, particularly within the 0–1.0 mg/kg range. However, when the BA dosage exceeded 1.0 mg/kg and reached 10.0 mg/kg, WGR, FI, and SR decreased. The FC gradually decreased across the 0–10.0 mg/kg concentration range. In conclusion, in the present study, the BA2 supplementation resulted in the highest FBW, WGR, and SR, while excessive bile acid supplementation may hinder shrimp growth.

### 3.2. The Effect of Adding BA to Feed on the Activity of Digestive Enzymes in the Gut Tract of P. vannamei

**Figure 1:**
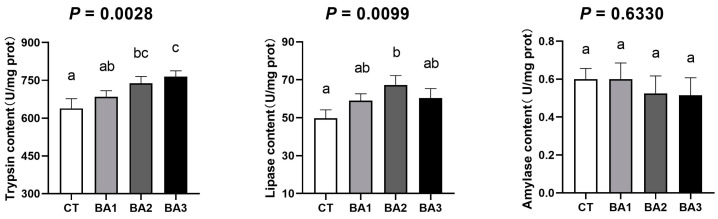
The effect of BA on the activity of digestive enzymes in the intestine of *P. vannamei*. Data were presented as mean ± SD, n = 3. ^a–c^ The values in the same row sharing different superscript letters are significantly different, as determined by one-way ANOVA and Turkey’s test (*p* < 0.05). CT—basal diet; BA1—basal diet supplemented with 0.1 mg/kg BA; BA2—basal diet supplemented with 1.0 mg/kg BA; BA3—basal diet supplemented with 10.0 mg/kg BA.

In order to explore the reasons for the improved growth performance of shrimp, gut enzyme activity was detected. In Figure 1, the effect of BA on the activity of digestive enzymes in the intestine of *P. vannamei* is presented. Different concentrations of BA promoted gut trypsin and lipase activities to varying extents. Specifically, trypsin activity increased with higher levels of BA supplementation. The trypsin activity in the BA2 and BA3 groups was significantly higher than in the CT group (*p* < 0.05). Among all groups, the BA2 group exhibited the highest lipase activity, which was significantly higher than that of the CT group (*p* < 0.05). The other two groups also showed an increasing trend in lipase activity compared to the CT group, although the differences were not statistically significant. The amylase results revealed that BA supplementation had little effect on amylase activity in the intestine, with no significant differences observed across the concentrations. In conclusion, bile acids enhanced the activities of trypsin and lipase in the gut of *P. vannamei*, thereby improving its digestive capacity.

### 3.3. The Effect of BA Added to Feed on Hepatopancreatic Antioxidant Enzyme Activity Capacity of P. vannamei

**Figure 2:**
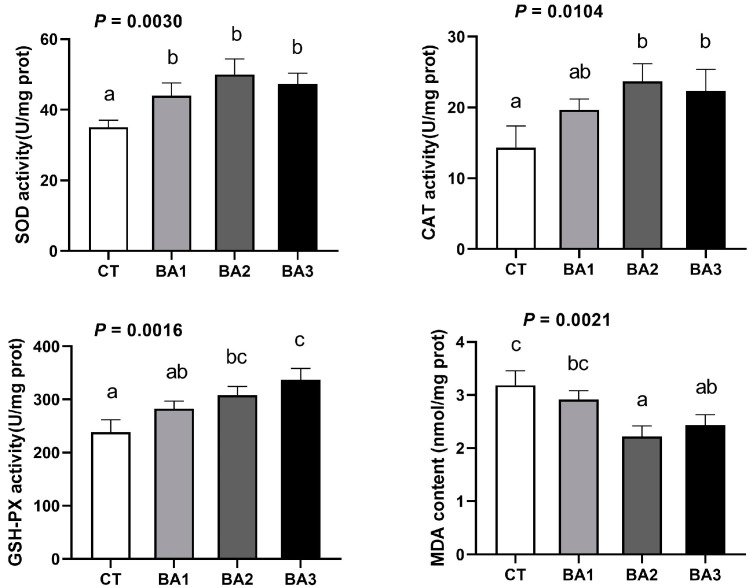
The effects of BA on antioxidant enzyme activity in hepatopancreas of *P. vannamei*. The data were presented as mean ± SD, n = 3. ^a–c^ Values in the same row sharing different superscript letters are significantly different, as determined by one-way ANOVA and Turkey’s test (*p* < 0.05). CT—basal diet; BA1—basal diet supplemented with 0.1 mg/kg BA; BA2—basal diet supplemented with 1.0 mg/kg BA; BA3—basal diet supplemented with 10.0 mg/kg BA.

Subsequently, we examined the effect of BA supplementation on the activity of antioxidant enzymes in the hepatopancreas of *P. vannamei*. In Figure 2, the effects of BA on antioxidant enzyme activity in hepatopancreas of *P. vannamei* are presented. BA addition positively affected the activities of SOD, CAT, and GSH-PX in the hepatopancreas to varying degrees. Notably, the SOD activity in the BA treatment group was significantly higher than in the CT group (*p* < 0.05), with the BA2 group exhibiting the highest SOD activity. The activity of CAT followed a similar trend to SOD, initially increasing before decreasing, while the BA2 and BA3 groups showed significantly greater CAT activity than the CT group (*p* < 0.05). Additionally, the activity of GSH-PX in the BA2 and BA3 groups was significantly higher than in the CT group, correlating with the increased BA supplementation (*p* < 0.05). MDA, a byproduct of lipid peroxidation, serves as an indirect indicator of tissue oxidative damage. The MDA content in the hepatopancreas decreased with BA supplementation, and the MDA levels in the BA2 and BA3 groups were significantly lower than in the CT group (*p* < 0.05). In conclusion, BA enhance the activity of antioxidant enzymes in the hepatopancreas of *P. vannamei* and reduce oxidative damage.

### 3.4. The Effect of Adding BA to Feed on the Expression of Gut Immunity-Related Genes in P. vannamei

**Figure 3:**
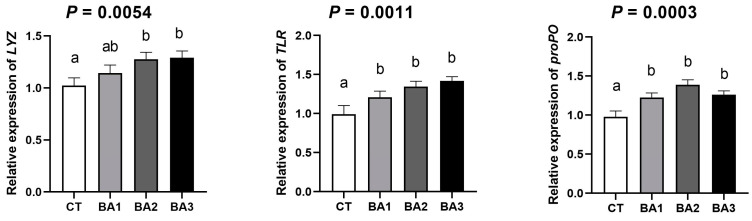
The effects of BA on gut immune-related gene expression in *P. vannamei*. The data were presented as mean ± SD, n = 3. ^a,b^ The values in the same row sharing different superscript letters are significantly different, as determined by one-way ANOVA and Turkey’s test (*p* < 0.05). CT—basal diet; BA1—basal diet supplemented with 0.1 mg/kg BA; BA2—basal diet supplemented with 1.0 mg/kg BA; BA3—basal diet supplemented with 10.0 mg/kg BA.

In order to explore the effect of BA supplementation on the immunity of *P. vannamei*, we measured the mRNA expression levels of key immune-related genes Lysozyme (*LYZ*), Toll-like receptor (*TLR*), and prophenoloxidase (*proPO*). As shown in Figure 3. Effects of BA on gut immune-related gene expression in *P. vannamei*, BA supplementation enhanced the mRNA expression of these immune genes in the shrimp gut. Both *LYZ* and *TLR* are critical genes involved in immune responses. Following treatment with different concentrations of BA, the expression levels of *LYZ* and *TLR* genes increased in a concentration-dependent manner. Specifically, the mRNA expression levels of *LYZ* in the BA2 and BA3 groups was significantly higher than in the CT group (*p* < 0.05), and the mRNA expression of TLR was also significantly elevated in the BA treatment groups compared to the CT group (*p* < 0.05). *ProPO*, an enzyme precursor primarily involved in immune defense and wound healing in invertebrates, showed significantly higher mRNA expression levels in the BA-treated groups than in the CT group (*p* < 0.05), with the highest level observed in the BA2 group. In conclusion, BA supplementation significantly enhanced the relative mRNA expression levels of the immune-related genes *LYZ*, *TLR*, and *proPO* in the gut of *P. vannamei*.

### 3.5. The Effects of Adding BA to Feeds on Gut Microorganisms of P. vannamei

**Figure 4:**
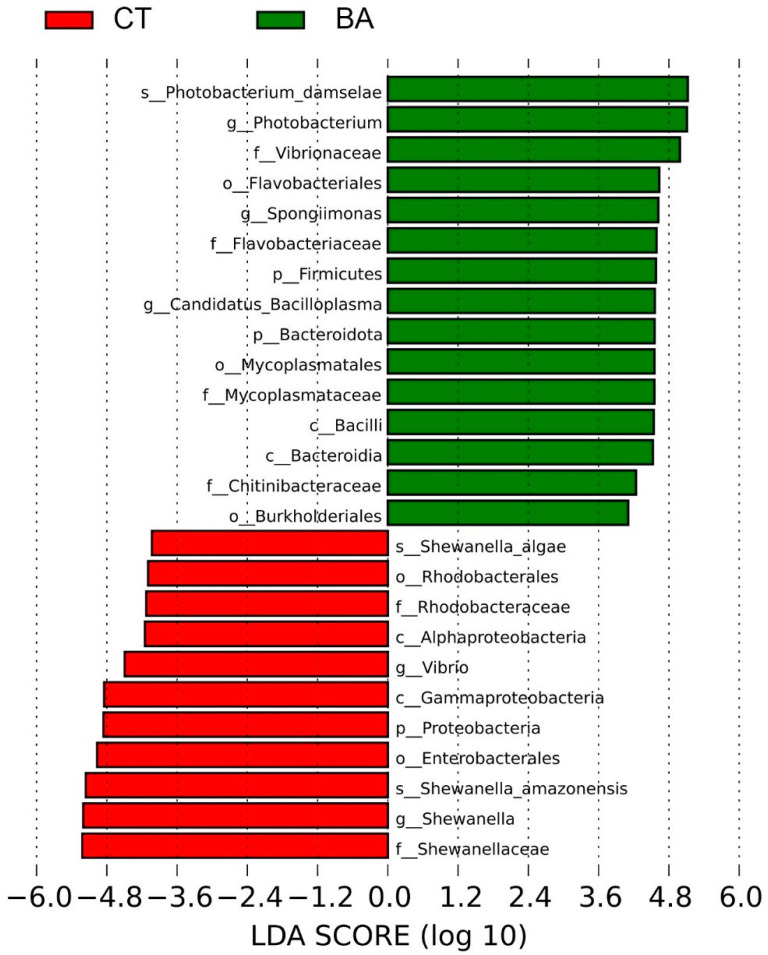
LEfSe analysis (LDA > 4). The data were presentedas mean ± SD, n = 3. ^a,b^ The values in the same row sharing different superscript letters are significantly different, as determined by one-way ANOVA and Turkey’s test (*p* < 0.05). CT—basal diet; BA2—BA, basal diet supplemented with 1.0 mg/kg BA.

The results above demonstrate that the BA2 group significantly improved body weight gain and survival rate, enhanced the antioxidant capacity of the hepatopancreas, and boosted both digestive function and immune capacity in the intestine of *P. vannamei*. In order to understand the differences in microorganisms in the gut of shrimp between the BA2 and CT groups, the gut microorganisms of the two groups were analyzed using LEfSe and screened for differential flora, with the screening criterion of LDA value > 4. A total of 3 phylums, 4 classes, 5 orders, 6 families, 5 genus g, and 3 species of microorganisms fulfilled the screening criterion (Figure 4). We can find that after the addition of 1.0 mg/kg BA, Photobacterium_damselae, *Photobacterium*, Vibrionaceae, Flavobacteriales, *Spongiimonas*, Flavobacteriaceae, Firmicutes, *Candidatus_ Bacilloplasma*, Bacteroidota, Mycoplasmatales, Bacilli, Bacteroidia, Chitinibacteraceae, and Burkhotderiales colonies increased significantly, making them the dominant flora in BA2. Shewanella algae, Rhodobacterales, Alphaproteobacteria, *Vibrio*, Gammaproteobacteria, Proteobacteria, Enterobacterales, Shewanella_amazonehsis, *Shewanella*, and Shewanellaceae flora were significantly reduced, and these were the dominant flora in the CT group.

**Figure 5:**
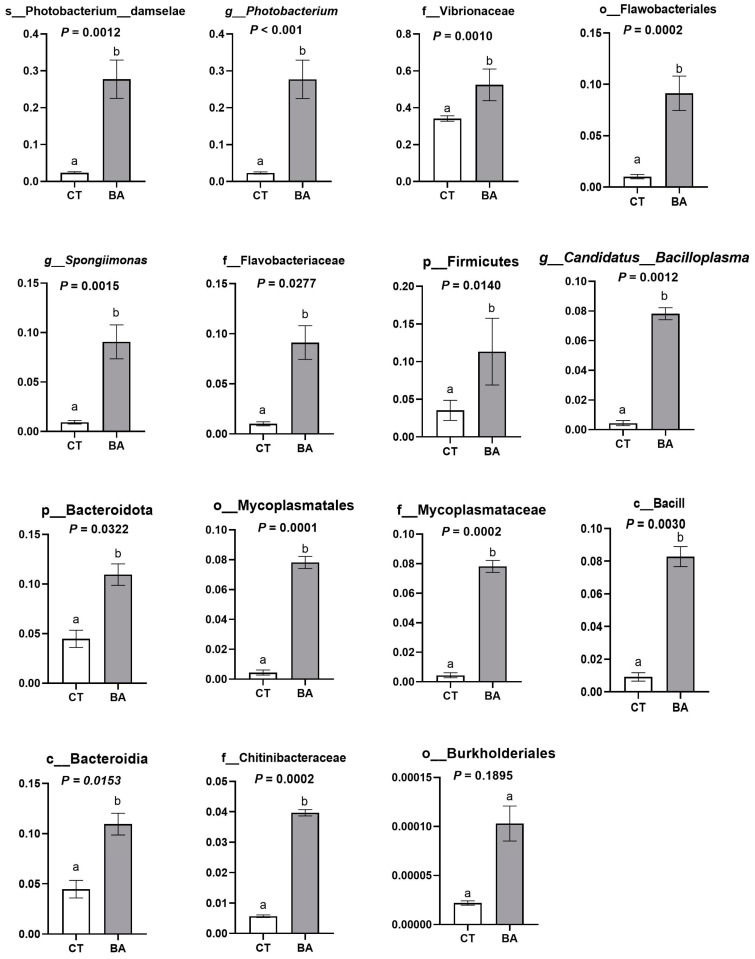
Differential analysis of dominant flora in the BA2 group. The data were presentedas mean ± SD, n = 3. CT—basal diet; BA2—BA, basal diet supplemented with 1.0 mg/kg BA.

By one-way ANOVA, we found that the content of Photobacterium_damselae, *Photobacterium*, Vibrionaceae, Flavobacteriales, *Spongiimonas*, Flavobacteriaceae, Firmicutes, *Candidatus_ Bacilloplasma*, Bacteroidota, Mycoplasmatales, Bacilli, Bacteroidia, and Chitinibacteraceae in the BA2 group was significantly higher than that in CT group (*p* < 0.05) (Figure 5).

## 4. Discussion

Bile acids (BA), with their amphiphilic structure, act as emulsifiers that facilitate feed emulsification and digestion by promoting fat micelle formation and enhancing the proteolytic breakdown of dietary proteins [29]. Research has indicated that supplementing feed with BA enhances growth performance and digestive enzyme activities in Japanese eels [36]. Similarly, the results of the present study found that supplementing the feed with 1.0 mg/kg of BA significantly improved shrimp growth performance, including final body weight, weight growth rate, average feed intake, and survival rate, as well as trypsin and lipase activities. In addition, it has been shown that adding BA to the feed increased the lipase activity without affecting the amylase activity in juvenile toothfish [22]. In the present study, we also found no significant effect of BA supplementation on the amylase activity of shrimp, which may be due to inefficient digestion and utilization of carbohydrates. In conclusion, the present study showed that BA could improve the growth performance and digestive enzyme activities of *P. vannamei*, which provides some theoretical basis for future research.

Under normal physiological conditions, the antioxidant defense system maintains the production and removal of reactive oxygen species (ROS). However, when this balance is disrupted, ROS accumulation can cause lipid peroxidation, damaging cell membranes and leading to the release of significant amounts of alanine aminotransferase (AST) and aspartate aminotransferase (ALT) into the bloodstream. Several studies have shown that BA not only reduces ROS directly, but also improves the antioxidant status of organisms by increasing the activity of antioxidant enzymes [37,38]. SOD, CAT, and GSH-PX are important enzymes in the antioxidant system of vertebrates [39]. MDA is the final product of lipid peroxidation, and its content is positively correlated with the degree of oxidative stress in organisms [40]. Relevant studies have shown that adding BA can enhance the antioxidant capacity of aquatic animals, and this effect has been confirmed in a variety of aquatic animals. These include largemouth bass [41], large yellow croaker [23], black seabream (*Acanthopagrus schlegelii*) [42], abalone (*Haliotis discus hannai*) [43], and Pacific White Shrimp [44]. In this study, we obtained similar results. The experimental group supplemented with BA showed more significant SOD, CAT, and GSH-PX activities, while the MDA content was significantly reduced. Therefore, the results indicated that appropriate BA could improve the antioxidant capacity of shrimp.

It is well known that, as a crustacean, shrimp do not have a specific immune system and can only rely on non-specific immunity to defend themselves against invading pathogens [45]. Additionally, the intestinal immune system is crucial to the shrimp’s immune system. Specifically, the intestine can fight against foreign pathogens from the surrounding environment [46], and intestinal epithelial cells can maintain intestinal homeostasis by mediating the expression of specific immune factors [47]. LYZ is a hydrolase with bactericidal properties, which is widely distributed in various biological fluids and tissues and has strong antibacterial activity [48]. TLRs are extremely important in the innate immune system. Previous studies have suggested that the interaction of innate immune defense and proinflammation may contribute to inflammatory diseases via TIR [49]. Prophenoloxidase (*proPO*) plays an important recognition and defense role in non-characteristic immune responses in crustaceans, protecting invertebrates from infection by harmful microorganisms [50]. In this study, the addition of BA increased the relative expression of the immune-related genes *LYZ*, *TLR*, and *proPO* in the gut of shrimp. Studies have shown that BA supplementation increased the mRNA expression level of intestinal lysozyme gene in largemouth bass [51], cholesterol supplementation (a precursor of BA synthesis) enhanced the activity of rainbow trout (*Oncorhynchus mykiss*) [52], and dietary BA supplementation increased the activity of lysozyme in the plasma of largemouth black sea bass [24] and the liver of black sea bream [42]. Studies have shown that when BA in diet is increased from 0 to 30 mg/kg, the expressions of TLR2 and TLR4 in the digestive glands of abalone are significantly decreased and then increased [43]. Therefore, it shows that the addition of BA can improve the intestinal immunity of shrimp and promote their healthy growth.

Animals have many active microbial communities in their gut that benefit the host by improving the immune response, promoting the digestion and absorption of nutrients, and maintaining homeostasis [53]. Gut microbiota can maintain the normal function of the gut, and in order to understand the relationship between the host and the gut microbiota, the gut microbiota has gradually become a focus of microbial research [54]. BA can regulate the composition of gut microorganisms [55]. The addition of 1.0 mg/kg significantly improved the growth performance, antioxidant capacity of hepatopancreas, digestive capacity, and immunity in the intestine of *P. vannamei*. Therefore, we focused on the intestinal differential microbes in the BA2 and CT groups. The LEFSe results showed that the majority of the dominant strains in the CT group belonged to the Proteobacteria, indicating that their abundance was decreased after the addition of BA. Although studies of BA’s effects on crustacean gut microbes have not been reported, studies on other aquatic animals, such as Chinese perch (*Siniperca chuatsi*) [56], *Haliotis discus hannai* [43], and tongue sole (*Cynoglossus semiliaevis*) [57], showed that supplementation with BA significantly changed the structural proportion of gut microbiota. Studies on the effects of BA in grass carp show that the addition of BA reduces the abundance of Proteobacteria in their gut microbes [27], which is the same as our research results. It can be seen that the richness of the microbial communities of Firmicutes and Bacteroidetes increased in the BA2 group. Firmicutes belong to the Gram-positive bacteria and contain most of the benign flora, such as Lactobacillus and Bacillus, which may have the ability to increase the immune capacity of the organism [58]. This may be one reason why the proper use of BA in the diet can improve the immunity of shrimp. Bacteroidetes can maintain the integrity of shrimp intestinal epithelial cells, enhance the tightness of connections, and promote the digestion and absorption of polysaccharide short-chain fatty acids [59]. Therefore, increasing the abundance of the gut microorganisms Firmicutes and Bacteroidetes may help promote the intestinal health of shrimp, and thus promote the growth and development of *P. vannamei*.

In summary, in this study, the supplementation of BA in the basic diet, especially the supplemental amount of 1.0 mg/kg, could improve the intestinal digestive ability, enhance the antioxidant capacity of hepatopancreas and intestinal immunity, improve the intestinal microbial community structure, and promote the growth of *P. vannamei*. However, there are some limitations in this study, such as insufficient in-depth research on the mechanism of impact on shrimp and single species of research object. In the future, we should further study the specific mechanism of BA affecting the growth of aquatic animals and explore its impact on other shrimp development stages and species.

## 5. Conclusions

In conclusion, the results of this study demonstrated that, among the BA supplementation levels ranging from 0.1–10 mg/kg to 1.0 mg/kg, BA effectively enhanced the growth performance, digestive ability, and antioxidant capacity of *P. vannamei*. Meanwhile, the addition of BA increased the mRNA expression levels of relevant immune genes in the intestine of shrimp. In addition, BA supplementation could change the microbial structure of the shrimp gut, promoting the growth of Firmicutes and Bacteroidetes, while significantly reducing the abundance of harmful Proteobacteria. Therefore, based on the results of this study, we recommend 1.0 mg/kg BA as the optimal addition to shrimp culture for promoting optimal growth, immune improvement, and intestinal health.

## Figures and Tables

**Table 1:** Shrimp basic feed ingredients and nutritional composition.

Ingredients	Content (%)
Fish meal	25.00
Soybean meal	20.00
Peanut meal	10.00
Canola meal	5.00
Shrimp shell meal	6.00
Flour	23.00
Fish oil	5.00
Microcrystalline cellulose	1.70
Phosphatidylinositol	1.50
Calcium bis	1.50
^1^ Vitamin premix	0.50
^2^ Mineral premix	0.50
Sodium chloride	0.20
Vitamin C ester	0.10
Nutrient components	
Crude protein	42.54
Crude lipid	9.63
Ash	7.23
Dry matter (DM, %)	Energy content (kcal/kg)
93.5	3300

^1^ Each kg of vitamin premix includes Vitamin B1 4 g, Vitamin B2 8 g, Vitamin B6 4.8 g, Vitamin B12 0.016 g, Vitamin E 16 g, Vitamin K 4 g, Niacin 28 g, Inositol 40 g, Calcium Pantothenate 16 g, Biotin 0.064 g, and Folic Acid 1.28 g. ^2^ Each kg of mineral premix includes magnesium sulfate monohydrate 12 g, calcium iodate 9 g, potassium chloride 36 g, copper methionine 1.5 g, zinc sulfate monohydrate 10 g, iron sulfate monohydrate 1 g, cobalt methionine 0.25 g, and sodium selenite 0.0036 g.

**Table 2:** Primer name and sequence.

Primer Name	Sequence (5′-3′)	Fragment Size (bp)	Accession Number
*β-actin*-F	GCCCTGTTCCAGCCCTCATT	944	AY486466.2
*β-actin*-R	ACGGATGTCCACGTCGCACT		
*LYZ*-F	GAAGCGACTACGGCAAGAAC	1069	XM_070138434.1
*LYZ*-R	AACCGTGAGACCAGCACTCT		
*TLR*-F	GACCATCCCTTTTACACCAGACT	4090	XM_070131812.1
*TLR*-R	CCTCGCACATCCAGGACTTTTA		
*proPO*-F	CAATGACCAGCAGCGTCTTC	2061	AF521948.1
*proPO*-R	CACGGAAGGAGGCGTATCAT		

*LYZ*—Lysozyme, *TLR*—Toll-like receptor, *proPO*—Prophenoloxidase.

**Table 3:** The effect of adding BA to feeds on the growth performance of *P. vannamei*.

	^3^ Group				
	CT	BA1	BA2	BA3	*p*-Value
^1^ IBW (g)	^2^ 1.22 ± 0.04 ^a^	1.21 ± 0.06 ^a^	1.21 ± 0.07 ^a^	1.21 ± 0.06 ^a^	0.9863
FBW (g)	13.75 ± 1.14 ^a^	14.11 ± 0.82 ^ab^	16.29 ± 0.95 ^b^	16.09 ± 0.89 ^ab^	0.0219
WGR (%)	1126.78 ± 93.50 ^a^	1165.84 ± 67.56 ^ab^	1346.56 ± 78.56 ^b^	1329.75 ± 73.31 ^ab^	0.0186
FI (g)	24.28 ± 1.22 ^a^	24.81 ± 0.86 ^a^	26.93 ± 1.26 ^a^	25.96 ± 1.14 ^a^	0.3235
FC	1.94 ± 0.1 ^a^	1.92 ± 0.07 ^a^	1.79 ± 0.08 ^a^	1.74 ± 0.08 ^a^	0.2832
SR (%)	86.67 ± 2.08 ^a^	89.33 ± 1.53 ^ab^	91.67 ± 1.53 ^b^	91.33 ± 1.15 ^b^	0.0180

^1^ IBW—initial body weight, FBW—final body weight, WGR—weight gain rate, FI—feed intake, FC—feed conversion ratio, SR—survival rate. ^2^ The data were presented as mean ± SD, n = 3. ^a,b^ The values in the same row sharing different superscript letters are significantly different, as determined by one-way ANOVA and Turkey’s test (*p* < 0.05). ^3^ CT—basal diet; BA1— basal diet supplemented with 0.1 mg/kg BA; BA2— basal diet supplemented with 1.0 mg/kg BA; BA3— basal diet supplemented with 10.0 mg/kg BA.

## Data Availability

The original contributions presented in the study are included in the article. Further inquiries can be directed to the corresponding author.
